# Comprehensive Comparison of Methods for Isolation of Extracellular Vesicles from Human Plasma

**DOI:** 10.1021/acs.jproteome.5c00149

**Published:** 2025-05-13

**Authors:** Patil Shivprasad Suresh, Qibin Zhang

**Affiliations:** † Center for Translational Biomedical Research, 14616 University of North Carolina at Greensboro, North Carolina Research Campus, Kannapolis, North Carolina 28081, United States; ‡ Department of Chemistry & Biochemistry, University of North Carolina at Greensboro, Greensboro, North Carolina 27402, United States

**Keywords:** plasma, extracellular vesicles, EV isolation, NTA, Simple Western, proteomics

## Abstract

Extracellular vesicles (EVs) are a vital component in cell–cell communication and hold significant potential as biomarkers and therapeutic carriers. Having a reproducible and simple EV isolation method for small volumes of human plasma is essential for biomarker discovery. Although combining multiple methods has been a recent trend in its ability to minimize contamination, it is not ideal for clinical specimens due to the large sample number and small sample volume. This study compared EVs isolated from 100 μL of plasma by nine commonly used methods based on different principles, including centrifugation, polymer precipitation, size exclusion, electrostatic interaction, and affinity enrichment. The isolated EVs were characterized by particle size and number using nanoparticle tracking analysis, purity, and contaminants using Simple Western and overall proteomic profiles using bottom-up proteomics. Despite the same EV enrichment principle, individual methods isolated EVs exhibited distinct characteristics, likely due to variations in the physicochemical properties of materials used and specific protocols. Overall, all of the methods evaluated are reproducible. MagNet and MagCap methods result in purer EVs with the narrowest size distribution and the highest proteome coverage but modest yield. This is the first report on isolating EVs from 100 μL of plasma using nine different methods with detailed characterization.

## Graphical abstract

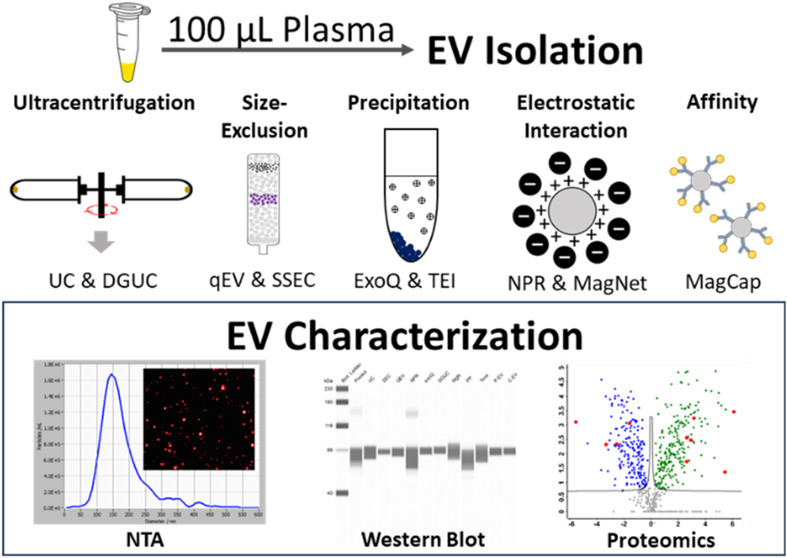

## 1 Introduction

EVs make up a diverse group of membrane-bound structures originating from various cells. They serve as vital mediators of intercellular communication, transporting cellular cargo that reflects the state of their cellular origin. Commonly, EVs fall into three main categories: apoptotic bodies (50–1000 nm), microvesicles (100–1000 nm), and exosomes (30–150 nm).^1^ All cell types secrete EVs, which are present in various body fluids, including urine, breast milk, and plasma.^2^ EVs can carry cell-specific cargo to recipient cells.^3^ They play critical roles in immune system activation and tolerance as well as in the progression of numerous diseases, such as cancer and type 1 diabetes,^4^ positioning them as promising targets and vehicles for therapeutic interventions.^5^ Additionally, EVs released during disease states can contain specific molecular composition of the disease, highlighting their potential as valuable biomarkers.^6,7^ The potential of EVs as biomarkers is a significant study area, with plasma-derived EVs being particularly intriguing due to their applicability in liquid biopsies.^8^ However, isolating EVs with high purity remains a complex and challenging task due to their small size and heterogeneity within biological fluids. As the International Society for Extracellular Vesicles (ISEV) emphasizes, the need for standardized isolation and quality assessment methods is urgent and of the utmost importance to advance fundamental research and biomarker discovery effectively.

Human plasma is a complex fluid containing abundant, soluble proteins, such as albumin, and various lipoproteins such as HDL, LDL, IDL, VLDL, and chylomicrons. Lipoproteins and EVs share similar characteristics such as density, size, and lipid content, with lipoproteins outnumbering EVs by an estimated 10^3^- to 10^6^-fold in plasma.^9,10^ This similarity and mass dominance complicate the distinction and isolation of EVs from lipoproteins, posing a significant challenge in EV research, mainly when EVs are used for biomarker discovery. Traditional methods like ultracentrifugation (UC) or density gradient ultracentrifugation (DGUC), particularly DGUC, can isolate EVs with minimal coenrichment of other plasma components. However, alternative techniques such as size exclusion chromatography and affinity methods are increasingly utilized for their efficiency in isolating EVs while minimizing the co-isolation of non-EV particles.^11^ Prior research indicates that achieving high purity in EV populations and minimizing the contamination of lipoproteins and other co-isolated particles necessitate employing multiple isolation techniques, especially when working with plasma.^12–14^ Several of these studies utilize a substantial volume of plasma, a practice that is impractical for most clinical diagnostic and biomarker discovery research applications, where only small volumes of clinical samples are available.

Many methods for isolating EVs from various samples have been developed by utilizing different principles. These include centrifugation,^15–17^ size exclusion chromatography (SEC),^18–20^ polymer precipitation,^21,22^ electrostatic interaction,^23–25^ and affinity enrichment.^26–29^ Centrifugation-based UC is predominantly used for EV enrichment, whereas DGUC is further employed to enhance the purity of EV populations from biological samples by separating particles based on their density.^30^ Conversely, SEC can differentiate particles by size, allowing larger EV particles to be eluted first, then smaller particles and soluble proteins to pass through the column.^31^ The mechanism of polymer precipitation reduces the solubility of EVs by forming a hydrophobic polymer layer around them, resulting in aggregation and formation of precipitate out of solution. Electrostatic interaction- and affinity enrichment-based methods isolate EVs by selectively targeting vesicles based on their surface chemistry. For instance, strong anion exchange magnetic resin electrostatically interacts with negatively charged vesicles, while affinity enrichment beads utilize specific recognition between bead-conjugated proteins and ligands on the EV surface. Particularly, MagCapture beads selectively isolate phosphatidylserine positive (PS+) EVs by binding bead-conjugated Tim4 protein to the PS on the EV surface.^32^ Few studies also attempted to evaluate the efficiency of multiple EV isolation methods based on the above-mentioned principles from different biological sources.^33–35^

In this study, we conducted a comparative analysis of nine different methods for their efficacy in isolating EVs from human plasma. Following the isolation, the EVs yield and size distribution were analyzed by nanoparticle tracking analysis. The particles’ purity was assessed by Simple Western with antibodies against canonical EV surface marker proteins and typical contaminants, and proteome coverage was explored by LC-MS/MS-based bottom-up proteomics. The primary objective was to identify reproducible and efficient methods for isolating EVs from small plasma volumes to facilitate reliable biomarker discovery.

## 2 Materials and Methods

### 2.1 Materials

Reagents/kits used for EV isolation were purchased from the following manufacturers/vendors: ultra-clear ultracentrifuge tubes (cat# C14293, Beckman Coulter), OptiPrep density gradient medium of 60% iodixanol (cat# D1556, Sigma), qEVsingle columns (cat# ICS-35, Izon Science), ExoQuick ULTRA kit (cat# EQULTRA-20A-1, System Biosciences), SmartSEC Single EV isolation kit (cat# SSEC200A-1, System Biosciences), Total exosome isolation kit (cat# 4484450, Invitrogen), Plasma/Serum Exosome Purification Mini-Kit (cat# 57400, Norgen Biotek), MagResyn SAX magnetic beads (cat# MR-SAX002, ReSyn Biosciences), and MagCapture exosome isolation kit PS Ver.2 (cat# 294-84101, Fujifilm/Wako Pure Chemical). Antibodies used in Simple Western analysis were purchased from the following companies: anti-CD9 (cat# 13174), anti-annexin A2 (cat# 8235S), and anti-HSPA8 (cat# 8444S) from Cell Signaling Technologies; anti-CD81 (cat# MAB46152), anti-albumin (cat# MAB1455), and anti-ApoA1 (cat# AF3664) from R&D Systems; anti-Alix (cat# NBP1-49701), anti-TSG101 (cat# NBP2-67884), and anti-ApoE3 (cat# MAB41442) from Novus Biologicals.

### 2.2 EV Isolation

As part of the clinical trial NCT03445234 that evaluate the effects of functional foods on the recovery of exercise-induced physiological stress,^36^ human blood was collected from healthy donors via venipuncture into BD K2EDTA vacutainer tubes and immediately centrifuged at 2000*g* for 10 min at 4 °C to collect plasma. Plasma samples were pooled and saved in a −80 °C freezer. Prior to analysis, pooled plasma was thawed at room temperature and then centrifuged at 3000*g* for 10 min at 4 °C. Subsequently, plasma EVs were isolated in triplicate with 100 μL plasma, utilizing nine distinct methods, as illustrated in Figure 1 and described in detail below (Sections 2.2.1–2.2.9). Immediately after EV isolation, particle yield and size were quantified using NTA. To minimize the impact of solvent heterogeneity on downstream EV analysis and ensure consistency in the final EV solvent and volume, all isolated EV samples were concentrated and buffer-exchanged using 10 kDa molecular weight cutoff filters (Amicon Ultra-0.5 mL, cat# UFC501096), normalizing the final volume to 100 μL PBS. This step removes residual polymers, salts, EV isolation reagents, and other contaminants that could interfere with downstream Simple Western and Proteomic analyses.

**Figure 1:**
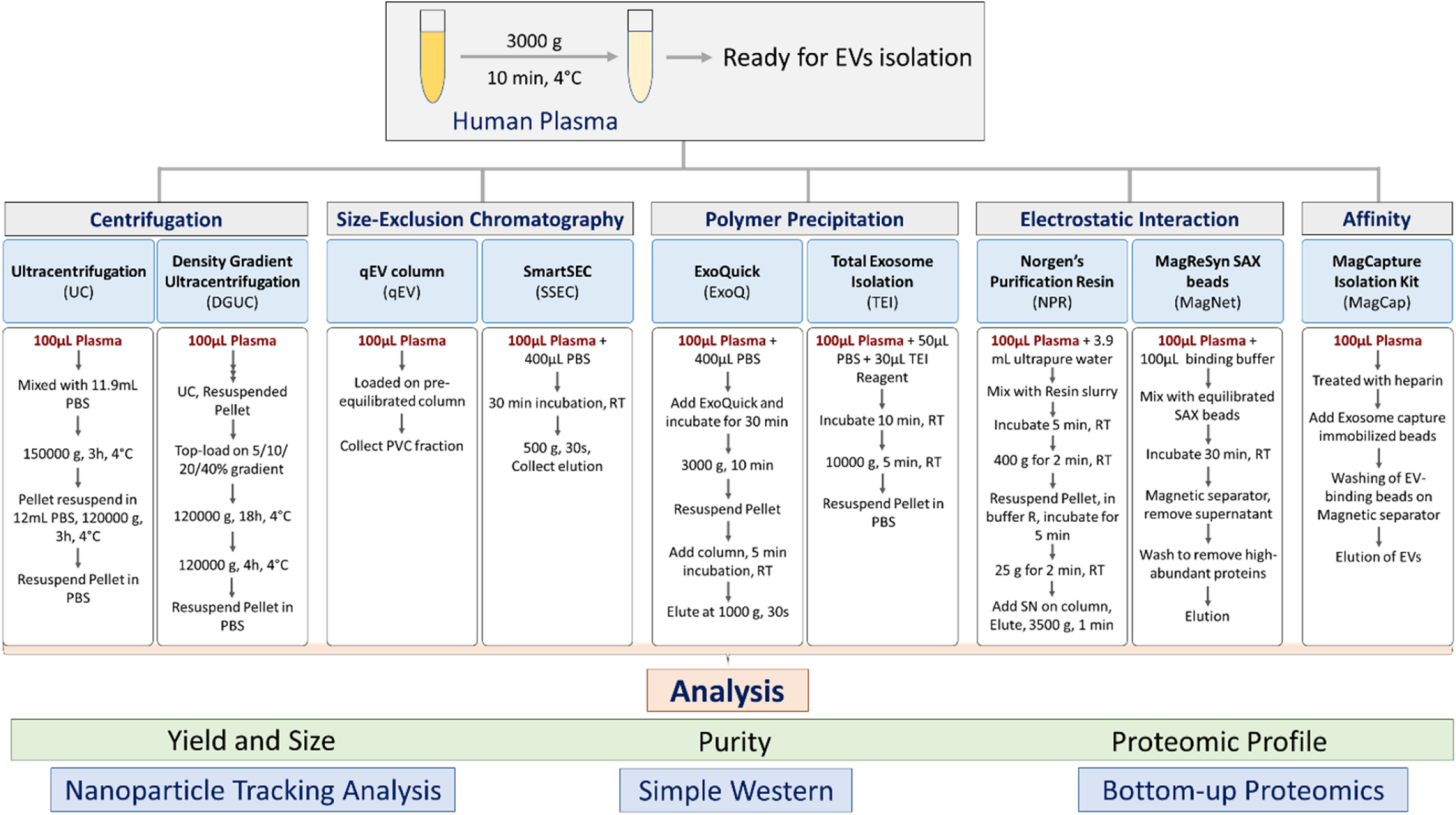
Schematic overview of the study design. EV isolation and analysis from human plasma were performed using different EV isolation methods. Each isolation was performed in triplicate.

#### 2.2.1 Ultracentrifugation (UC)

Plasma samples (100 μL) were diluted with 11.9 mL of phosphate-buffered saline (PBS, pH 7.4) and transferred to ultracentrifuge tubes (Ultra-Clear, Beckman Coulter). The samples were subjected to ultracentrifugation using a Sorvall WX 80 Ultracentrifuge with TH-641 swinging-bucket rotor (Thermo Scientific) at 1,50,000*g* for 3 h at 4 °C to pellet EVs and larger particles. The supernatant was carefully aspirated, and the resulting pellet was resuspended in 12 mL of PBS. A second centrifugation step was performed at 1,20,000*g* for 3 h at 4 °C to purify the EVs further. The final pellet was resuspended in 100 μL of PBS for NTA analysis and then stored at −20 °C until further analysis.

#### 2.2.2 Density Gradient Ultracentrifugation (DGUC)

Iodixanol (OptiPrep, Sigma-Aldrich) density gradient was prepared by layering 5, 10, 20, and 40% (*w/v*) iodixanol solutions in PBS sequentially in a 13.2 mL ultracentrifuge tube (Ultra-Clear, Beckman Coulter). The EV pellet obtained from ultracentrifugation (Section 2.2.1) was resuspended in PBS and carefully overlaid onto the gradient. The samples were centrifuged at 1,20,000*g* for 18 h at 4 °C in a TH-641 rotor (Sorvall WX 80 Ultracentrifuge). Following centrifugation, the upper 6 mL fraction, containing EVs, was collected, diluted with an equal volume of ice-cold PBS, and centrifuged at 1,20,000*g* for 4 h at 4 °C. The final EV pellet was resuspended in 100 μL of PBS for NTA analysis and then stored at −20 °C until downstream analyses.

#### 2.2.3 qEV Column (qEV)

The plasma EVs were isolated using a qEVsingle 35 nm column (Izon Science) according to the manufacturer’s protocol. Briefly, the column was pre-equilibrated with 6 mL of PBS (pH 7.4). Plasma (100 μL) was loaded onto the column and eluted with 1.4 mL of PBS. The first 0.7 mL (void volume) was discarded, and the subsequent 0.7 mL fraction, enriched in EVs, was collected. The eluted EVs were immediately analyzed with NTA and stored at −20 °C until further downstream analyses.

#### 2.2.4 SmartSEC (SSEC)

The SmartSEC Single EV Isolation System (System Biosciences) was used for plasma EV isolation. Plasma (100 μL) was diluted with 400 μL of PBS and loaded onto a pre-washed SmartSEC column. The sample was incubated at room temperature for 30 min with gentle rotation (10 rpm) to facilitate EV-beads interaction. Following incubation, EVs were eluted by centrifugation at 500*g* for 30 s. The eluate (500 μL) was collected, immediately analyzed with NTA, and stored at −20 °C until further downstream analyses.

#### 2.2.5 ExoQuick (ExoQ)

EVs were isolated using the ExoQuick ULTRA Exosome Precipitation Solution (System Biosciences). Briefly, 100 μL of plasma was mixed with 30 μL of ExoQuick precipitation reagent and incubated on ice for 30 min. The mixture was centrifuged at 3000*g* for 10 min at 4 °C to pellet EVs. The supernatant was discarded, and the pellet was resuspended in 200 μL of Buffer B (provided in the kit). Subsequently, 200 μL of Buffer A (supplied in the kit) was added, and the mixture was loaded onto a pre-washed purification column (provided in the kit). After incubating at room temperature for 5 min with gentle, continuous shaking, the column was centrifuged at 1000*g* for 30 s to collect purified EVs. The eluted EVs (400 μL) were immediately analyzed with NTA and stored at −20 °C until further downstream analyses.

#### 2.2.6 Total Exosome Isolation (TEI)

The Total Exosome Isolation Reagent (Thermo Fisher Scientific) was used following the manufacturer’s protocol. Plasma (100 μL) was mixed with 50 μL of PBS, followed by the addition of 20 μL of the exosome precipitation reagent (provided in the kit). The mixture was vortexed and incubated at room temperature for 10 min. Then, plasma EVs were centrifuged at 10,000*g* for 5 min at room temperature. The supernatant was carefully removed, and the pellet was resuspended in 100 μL of PBS, immediately analyzed with NTA, and then stored at −20 °C for further analysis.

#### 2.2.7 Norgen’s Purification Resin (NPR)

EVs were isolated using the Plasma/Serum Exosome Purification Kit (Norgen Biotek). Plasma (100 μL) was diluted in 3.9 mL of nuclease-free water, and 100 μL of ExoC buffer (provided in the kit) was added. The mixture was gently mixed, followed by the addition of 200 μL of Slurry E resin (supplied in the kit). After a 5 min incubation at room temperature, the sample was centrifuged at 400*g* for 2 min. The supernatant was discarded, and 200 μL of ExoR buffer (provided in the kit) was added to the resin. Following another 5 min of incubation, the sample was centrifuged at 25*g* for 2 min. The supernatant was transferred to a mini-filter spin column (provided in the kit) and centrifuged at 3500*g* for 1 min to collect purified EVs. The eluted EVs (200 μL) were immediately analyzed with NTA and stored at −20 °C until further downstream analyses.

#### 2.2.8 MagReSyn SAX Beads (MagNet)

MagReSyn SAX (strong anion exchange) beads (ReSyn Biosciences) were washed with equilibration/wash buffer containing 50 mM Bis-Tris (pH 6.5) and 150 mM NaCl. Plasma (100 μL) was mixed with an equal volume of EV binding buffer (100 mM Bis-Tris Propane, pH 6.3, 150 mM NaCl) and incubated with pre-equilibrated SAX beads for 30 min at room temperature with gentle agitation. Following this incubation, the flow-through containing unbound plasma proteins was removed by using a magnetic separator. The SAX beads were washed three times with equilibration/wash buffer to deplete highly abundant plasma proteins. Finally, the plasma EVs were eluted with 100 μL of elution buffer (25 mM Bis-Tris Propane, pH 6.5, 1 M NaCl, and 0.1% Tween 20). The eluted EVs were immediately analyzed with NTA and stored at −20 °C until further downstream analyses.

#### 2.2.9 MagCapture Isolation Kit (MagCap)

The MagCapture Exosome Isolation Kit PS (Fujifilm Wako) was used for immunoaffinity-based plasma EV isolation by following the manufacturer’s protocol. 100 μL of Plasma was pretreated with 5 U of heparin to reduce nonspecific binding. The sample was then incubated with exosome capture-immobilized magnetic beads and exosome binding enhancer (provided in the kit) for 1 h at 4 °C on a rotating mixer (500 rpm). Following incubation, the beads were washed three times using a washing buffer (provided in the kit) and a magnetic separator. EVs were eluted by adding 50 μL of elution buffer (provided in the kit) twice. The eluted EVs were immediately analyzed with NTA and stored at −20 °C until further downstream analyses.

### 2.3 Nanoparticle Tracking Analysis

Upon isolation, an aliquot of the EV samples underwent a 1000-fold dilution with PBS, and it was immediately characterized for the particle number and size on a ZetaView Quatt (Particle Metrix) instrument in scatter mode. Parameters were set as follows: camera sensitivity at 80, shutter at 100, minimum brightness of particles at 30, trace length at 7, and minimum and maximum area at 10 and 1000 nm, respectively. Each sample was analyzed in triplicate with the same settings, and the mean and standard deviation of the three replicates were used to plot the particle size distribution against the number of particles per mL.

### 2.4 Simple Western Analysis

The EV protein markers were examined using a fully automated Western blot system, the ProteinSimple Jess Simple Western instrument (Bio-Techne), following the manufacturer’s guidelines. For each of the nine methods tested, 20 μL of EV from each triplicate sample preparation was pooled for immunoassay and total protein assay. Additionally, all 27 samples from each replicate of each EV isolation method were analyzed for the CD9 protein marker.

For Simple Western analysis, 3 μL of EVs were combined with 0.5 μL of 5X RIPA, sonicated for 5 min, and then incubated on ice for 15 min. The lysed EV samples were mixed with fluorescent 5X master mix provided by the manufacturer containing 200 mM DTT, heated at 95 °C for 5 min, and kept on ice. Electrophoresis was performed using a 12–230 kDa Jess Separation Module (SM-W004) on a 25-capillary cartridge with chemiluminescence detection. The specific primary antibodies and their respective secondary antibodies were used to characterize CD9, CD81, Alix, annexin A2, HSPA8, TSG101, albumin, ApoA1, and ApoE3. Data analysis was performed by using Compass software (version 6.3.0). Total protein quantification was carried out using the total protein detection module for chemiluminescence (DM-TP01), and a RePlex Module RP-001 was included in each run to quantify loading. The RePlex Module facilitated the removal of primary and secondary antibodies in a RePlex assay, enabling total protein determination in a single run.

### 2.5 LC-MS/MS Proteomics of Isolated EVs

The EV samples were digested by using a modified S-Trap protein digestion protocol. Briefly, EVs (equivalent to 3 μL of plasma-derived EVs, diluted up to 50 μL in PBS) and 50 μL of 2X lysis buffer (10% SDS, 100 mM TEAB pH 8.5) were mixed and ultrasonicated for 10 min, to which 2 μL of reducing buffer (500 mM TCEP) was added and incubated at 55 °C for 20 min. Then, 2 μL of alkylator (500 mM IAA) was added, and the mixture was incubated at room temperature for 20 min, after which 5 μL of acidifier (30% phosphoric acid) was added. The mixture was vortexed, followed by the addition of 200 μL of binding/wash buffer (100 mM TEAB in 90% methanol). Then, the EV sample was applied to an S-Trap micro column (cat# C02-micro-80, ProtiFi) and centrifuged at 4000*g* for 30 s to trap proteins. The column was washed with 150 μL of binding/wash buffer and centrifuged at 4000*g* for 30 s, and the washing was repeated thrice, then centrifuged for 1 min at 4000*g* to remove additional traces of binding/wash buffer altogether. After protein digestion with 20 μL of digestion buffer (50 mM TEAB containing 1 μg of trypsin/LysC) and incubation for 3 h at 47 °C, the digested peptides were eluted with elution buffers (50 mM TEAB in water, 0.2% Formic Acid, and 50% ACN in water) by centrifuging at 4000*g* for 1 min after each addition.

The digested peptides were loaded onto EvoTip trap columns (Evosep, cat# EV2013) with the following procedures: washing the EvoTips with 20 μL of solvent B (acetonitrile with 0.1% formic acid), conditioning with 2-propanol, and equilibrating with 60 μL of solvent A (water with 0.1% formic acid). After the samples were loaded, the tips were washed twice with 60 μL of solvent A, followed by a final wash with 100 μL of solvent A for 10 s to ensure the tips did not dry out. All of these steps were performed in a centrifuge at 800*g* for 60 s.

The peptides on the EvoTips were then separated using an 8 cm × 150 μm column packed with 1.5 μm C18 beads (EV1109) on an Evosep One LC system (Evosep, Denmark) at a flow rate of 1 μL/min, using the 21 min gradient, 60 samples per day method. Peptide elution was accomplished within 35% solvent B. Eluted peptides were detected in positive ion mode using an Orbitrap Ascend Tribrid Mass Spectrometer (Thermo Fisher). Mass spectra were acquired within the range of 380 to 985 *m/z* at a mass resolution of 60 k (at 200 *m/z*), followed by data-independent acquisition (DIA) MS/MS with a mass isolation window of 10 *m/z* and a mass resolution of 30 k (at 200 *m/z*). Other critical settings on the Ascend included a normalized HCD collision energy of 25, a normalized AGC target of 200% for the MS1 scan and 100% for the DIA scan, and an ion injection time of 100 ms for the MS1 scan and 40 ms for the DIA scan.

### 2.6 Proteomics Data Analysis

The raw LC-MS/MS data were processed using DIA-NN (version 1.8.1).^37^ Protein identification utilized the Human database downloaded from Uniprot on June 07, 2024. The analysis featured several specific configurations, including a FASTA digest for library-free search and library creation and the application of deep learning algorithms for predicting spectra and retention times. Key parameters were set as follows: mass accuracy at 15.0, MS1 accuracy at 20.0, and scan window at 4. Trypsin/P was specified as an enzyme, allowing for one missed cleavage. Cysteine residues had carbamidomethyl set as a fixed modification, and the match between runs (MBR) feature was enabled for better data alignment. Protein inference was conducted on genes using a neural network classifier in single-pass mode, and quantification was fine-tuned for high-accuracy LC. Additionally, cross-run normalization was adjusted for retention time-dependent dynamics, and library profiling utilized smart profiling techniques. All other parameters used were their default settings.

The output files generated by DIA-NN were processed using Perseus software (version 1.6.14.0).^38^ Data were filtered to keep those proteins with >70% valid values in all samples. The data then underwent log2 transformation, followed by normalization via the width adjustment algorithm. Missing values were imputed by random numbers from a normal distribution. The statistically significant protein alterations were identified using a multiple-sample test with a permutation-based false discovery rate (FDR) threshold of <0.05 and an S0 value of <0.05. Further data exploration included hierarchical clustering (with Z-score normalization), volcano plot visualization, and principal component analysis (PCA).

## 3 Results

We aimed to evaluate various EV enrichment methods for their effectiveness in the reproducible isolation of high-yield plasma EVs. Given the limited volume of plasma samples in clinical biobanks, developing a high-throughput EV isolation protocol for small sample volumes is particularly beneficial. To this end, we used previously frozen human plasma as our starting material, specifically 100 μL of thawed plasma, for EV enrichment. With the recent surge in EV research interest, many commercial products have emerged to address the rapid isolation of EVs. We evaluated nine EV isolation methods predominantly used by EV researchers, including centrifugation-based UC and DGUC; size exclusion chromatography-based qEV and SSEC, polymer precipitation-based ExoQ and TEI, electrostatic interaction-based strong anion exchange resin (MagNet) and Norgen’s proprietary silicon carbide resin (NPR), and Tim4-PS affinity-based MagCap. EV isolation was performed according to the manufacturer’s instructions, then isolated EVs were characterized for their size and yield by NTA, purity by Simple Western, and proteomic profile by bottom-up proteomics (Figure 1).

### 3.1 Isolation Methods Impact Particle Size Distribution and Yield

NTA analysis demonstrated notable differences in EV size distributions among isolation methods (Figure 2), with average particle sizes ranging between 90 and 117 nm. Affinity-based (MagCap), electrostatic interaction-based methods (NPR and MagNet), and DGUC yielded the narrowest size distributions. In contrast, polymer precipitation-based methods (TEI and ExoQ) and size exclusion chromatography (qEV and SSEC), along with UC and ExoQ, exhibited broader distributions, including a substantial proportion of particles >200 nm. As proposed by Huang et al., EVs with a lipid bilayer are unlikely to be <30 nm; thus, particles below this threshold were classified as non-EV entities (e.g., lipoproteins, exomeres, or protein aggregates), contributing to background granularity.^39^ Among the methods, ExoQ and TEI contained ∼4 and ∼2% of particles <30 nm, respectively, while other methods showed <1%. Although qEV is optimized for isolating particles within the 35–350 nm range, detecting <30 nm particles may reflect adherence or co-elution with EVs.

**Figure 2:**
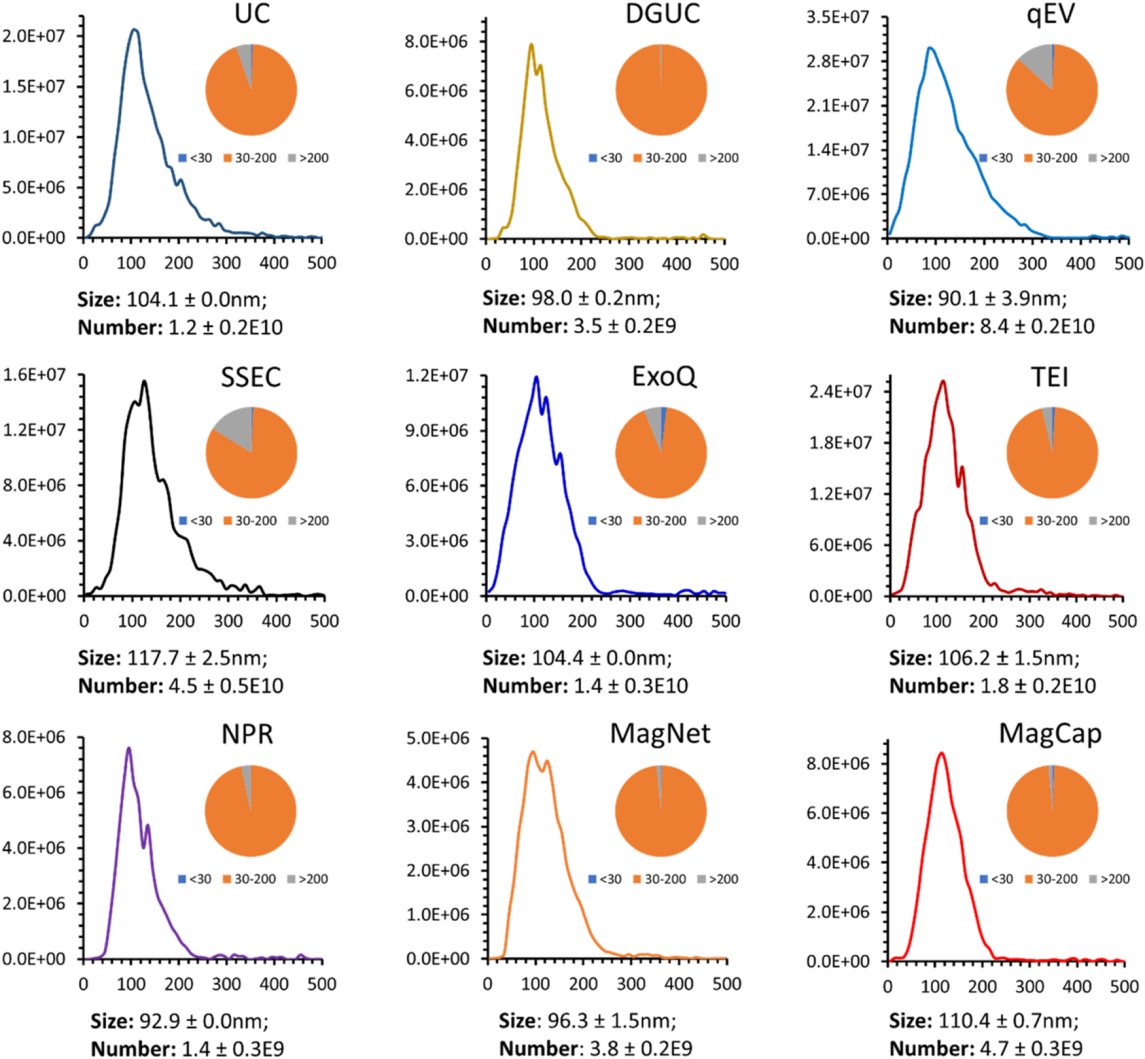
NTA-based characterization of plasma-derived EVs. Size distribution profiles of EVs isolated using nine different EV isolation methods (UC, DGUC, qEV, SSEC, ExoQ, TEI, NPR, MagNet, and MagCap) and the particle size distribution pie chart of each method. Blue color, non-EV particles <30 nm; orange color, small EV 30–200 nm; and gray color, large EV >200 nm sized particles. Measurements were based on three preparation replicates.

The yield of isolated particles varied across methods, with a general trend of a lower yield corresponding to narrower size distributions. MagCap, MagNet, NPR, and DGUC yielded a total of ∼1.4 to 4.7 E9 particles from 100 μL of plasma, representing an 8- to 17-fold reduction compared to UC, qEV, and SSEC, which enriched ∼1.2 to 8.4 E10 particles. Yield-wise, qEV enriched more EVs from 100 μL with ∼15% of large EVs, followed by SSEC with ∼17%. For the two polymer-precipitation-based methods, TEI had a slightly higher yield than ExoQ (1.8 E10 vs 1.4 E10 particles), likely because ExoQ is a 2-in-1 method with column separation following the initial polymer precipitation. Similarly, the yield comparison between UC (1.2 E10) and DGUC (3.5 E9), in which DGUC further separates EVs by density gradient (1.08–1.19 g/mL), followed by the initial ultracentrifugation step.

All nine methods showed high reproducibility, with coefficients of variation (CVs) for particle size and yields below 20%. Notably, the more selective methods (MagCap, MagNet, NPR, DGUC) predominantly enriched small EVs (30–200 nm), yielding more homogeneous populations compared to nonspecific methods (UC, qEV, SSEC). As expected, increased selectivity correlated with a reduced particle yield.

### 3.2 Purity Assessment by Measuring Classical EV Markers and Contaminants

EVs isolated via each method were evaluated for purity by analyzing canonical EV protein markers, including CD9, Alix, Annexin A2, HSPA8, TSG101, and contaminants like albumin, ApoA1, and ApoE3 using a capillary-based Simple Western immunoassay. As depicted in Figure 3A (full images of Simple Western analysis in Supplementary Figure S1), all isolation methods exhibited significant enrichment of EV markers relative to neat plasma, confirming efficient EV recovery. CD81, however, was selectively detected in EVs isolated by NPR, MagNet, and MagCap. Notably, despite the presence of CD81, the intensities of all other markers were lower in EVs isolated using NPR. This reduced intensity likely can be attributed to the lowest number of EVs isolated by this method, as indicated by NTA. Comparative analysis of equal-volume-loaded samples revealed superior marker band intensities in qEV, SSEC, ExoQ, MagNet, and MagCap isolates, suggesting a higher EV purity with these methods.

**Figure 3:**
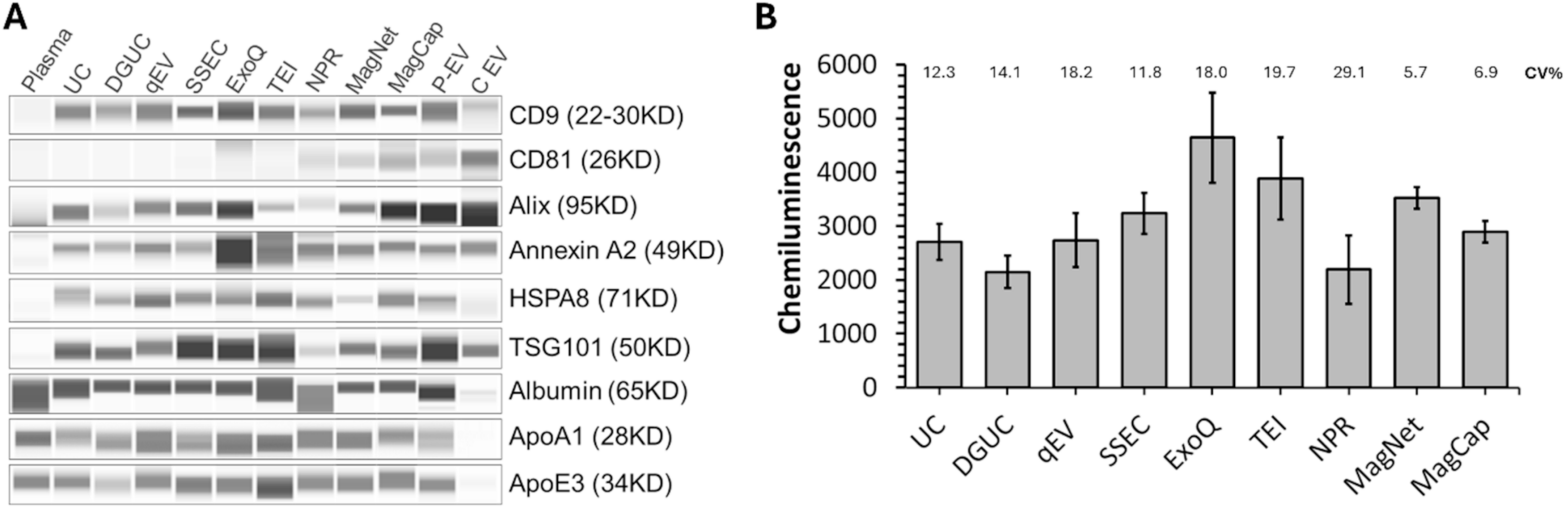
Purity assessment of EVs isolated by nine methods using Simple Western. (A) Comparison of EV markers and contaminant proteins in the nine EV isolation methods. Sample loading was normalized to the same starting plasma volume. Neat Plasma, HansaBioMed plasma EV (P-EV), and cell EV (C EV) standards were used as controls. (B) Reproducibility of EV preparation by measuring CD9 (*n* = 3, mean ± SD and CV%).

Another critical aspect of assessing the purity of isolated EVs is evaluating contamination introduced by highly abundant proteins (HAP). Contaminant analysis revealed a marked depletion of plasma-derived proteins, e.g., albumin, apolipoprotein A-I (ApoA1), and apolipoprotein E3 (ApoE3) across all isolation protocols compared to neat plasma (Figure 3A). However, among the nine EV isolation methods, albumin contamination was higher in the UC, TEI, and NPR methods. At the same time, DGUC isolates exhibited the most effective removal of apolipoproteins, with the ApoE3 reduction exceeding that of ApoA1. This disparity likely stems from density-dependent separation efficacy: ApoE3-associated LDL/VLDL (density: 1.006–1.063 g/mL) are more readily separable from EVs (1.10–1.21 g/mL) than ApoA1-bound HDL (1.063–1.21 g/mL).^40^

Reproducibility was assessed via triplicate CD9 chemiluminescence measurements (Figure 3B). EVs isolated by ExoQ, TEI, and MagNet showed elevated CD9 signals but exhibited higher inter-replicate variability (CV: ∼18–29%), consistent with NTA-derived particle count fluctuations. In contrast, MagCap and MagNet isolates demonstrated robust reproducibility (CV: <7%), underscoring their methodological consistency.

### 3.3 Proteomic Analysis Revealed Differences in EV Populations Isolated by Each Method

To evaluate whether isolation methods influence the molecular composition of plasma EVs, we performed LC-MS/MS analysis of tryptic digests from EVs isolated by each method. DIA-NN (peptide spectra library-free mode) identified >570 proteins across all methods, with proteomic coverage variability primarily attributed to isolation methodology rather than EV enrichment principles. MagNet (785 proteins) and MagCap (778 proteins) achieved the highest proteome coverage, likely due to their superior depletion of high-abundance plasma proteins via bead surface interactions. Size exclusion chromatography and polymer precipitation methods yielded comparable protein counts. NPR exhibited the lowest coverage (570 proteins; Figure 4A), consistent with its poor particle recovery and attenuated EV marker signals in Simple Western assays. The control samples, including neat plasma and HansaBioMed plasma EV and cell EV, identified 634, 723, and 767 proteins, respectively. The identified proteins from each EV isolation method are provided in Table S1. Overall, the CVs for the EV proteome coverage of all analyzed samples were less than 20%, except for the NPR at 23% (Figure 4B). Unsupervised principal component analysis (PCA) revealed method-specific clustering (PC1 and PC2: 44.4% variance; Figure 4C), with negligible intramethod batch effects (triplicate concordance). The hierarchical clustering also validates the clustering of each EV isolation method group (Figure S2). Neat plasma and reference EVs (HansaBioMed plasma/cell EVs) diverged markedly along PC1, whereas PC2 separated NPR and ultracentrifugation (UC and DGUC)-derived EVs from other methods.

**Figure 4:**
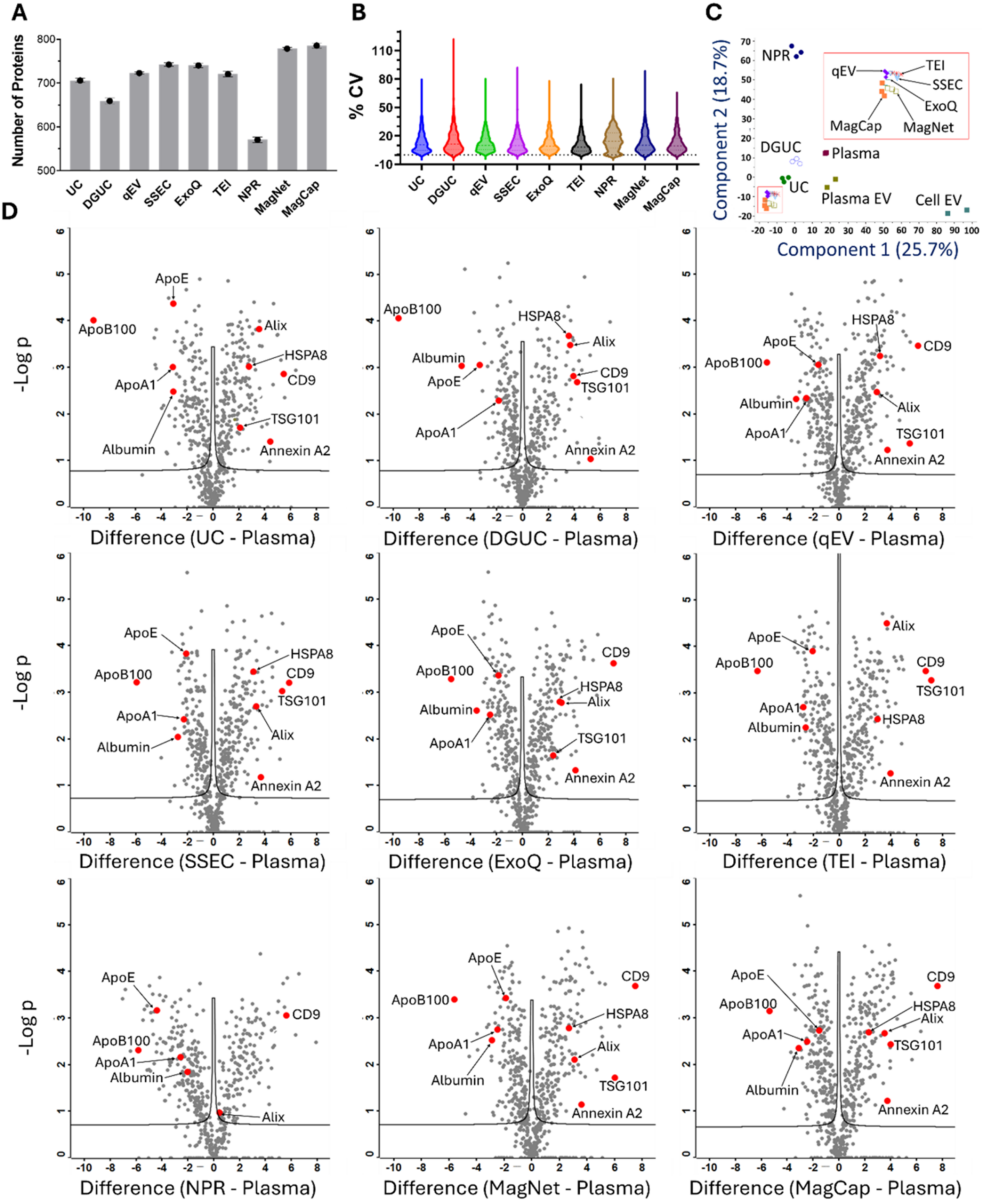
Proteomic characterization of different EV preparations. (A) Number of EV proteins identified. (B) CV% of each EV enrichment method. (C) PCA scores plot showing clustering and similarity between each EV isolation method. Neat plasma, plasma EV, and cell EV standards were used as controls. (D) Volcano plot showing significantly enriched and depleted proteins in each EV isolation method to neat plasma. Student’s t-test, with FDR and S0 < 0.05.

Proteomic data correlated well with Simple Western results, as validated by volcano plots quantifying EV marker enrichment and contaminant depletion relative to plasma (Figure 4D). CD9 showed maximal enrichment in ExoQ, MagNet, and MagCap (∼7-fold, log2 scale), then qEV, SSEC, TEI, and NPR (∼6-fold), followed by UC and DGUC (∼4-fold). In contrast, TSG101 was the most enriched (∼6-fold) in qEV, TEI, and MagNet, followed by MagCap, DGUC, and SSEC (∼4-fold), with only ∼2-fold enrichment in ExoQ and UC. Albumin depletion was most efficient in DGUC (∼5-fold) and qEV (∼4-fold), while the rest had ∼3-fold depletion. Meanwhile, lipoprotein contaminants (ApoA1/ApoE/ApoB100) exhibited method-dependent clearance. ApoA1 all had ∼2 to 3-fold depletion, but ApoE was depleted most in DGUC and NPR (∼4-fold), while the rest of the methods had ∼2-fold depletion. In addition to ApoA1 and ApoE, another lipoprotein, ApoB100, a significant component of VLDL, also depleted in all EV isolation methods, with the most depletion in the two centrifugation-based methods, UC and DGUC (∼10-fold), while the rest of the methods had ∼6-fold depletion. Proteomic analysis provides critical insights into EV purity by quantifying EV-enriched markers and contaminant depletion. High-purity isolates (e.g., MagNet/MagCap) exhibit the maximal enrichment of canonical EV markers (CD9, TSG101) with fold-changes >6; significant depletion of plasma contaminants (albumin, ApoB100) >5-fold reduction.

To further evaluate the efficiency of the plasma EV isolation methods, we compared our data with the ExoCarta database regarding EV-associated proteins. Method efficacy was further assessed against the ExoCarta database. All nine methods enriched >63% EV-associated proteins. The Venn diagram in Figure 5 showed that the EVs from MagNet- and MagCap methods contain ∼69 and 68% EV-associated proteins, respectively. Among them, 60 proteins belonged to the top 100 EV marker proteins listed in the ExoCarta database.

**Figure 5:**
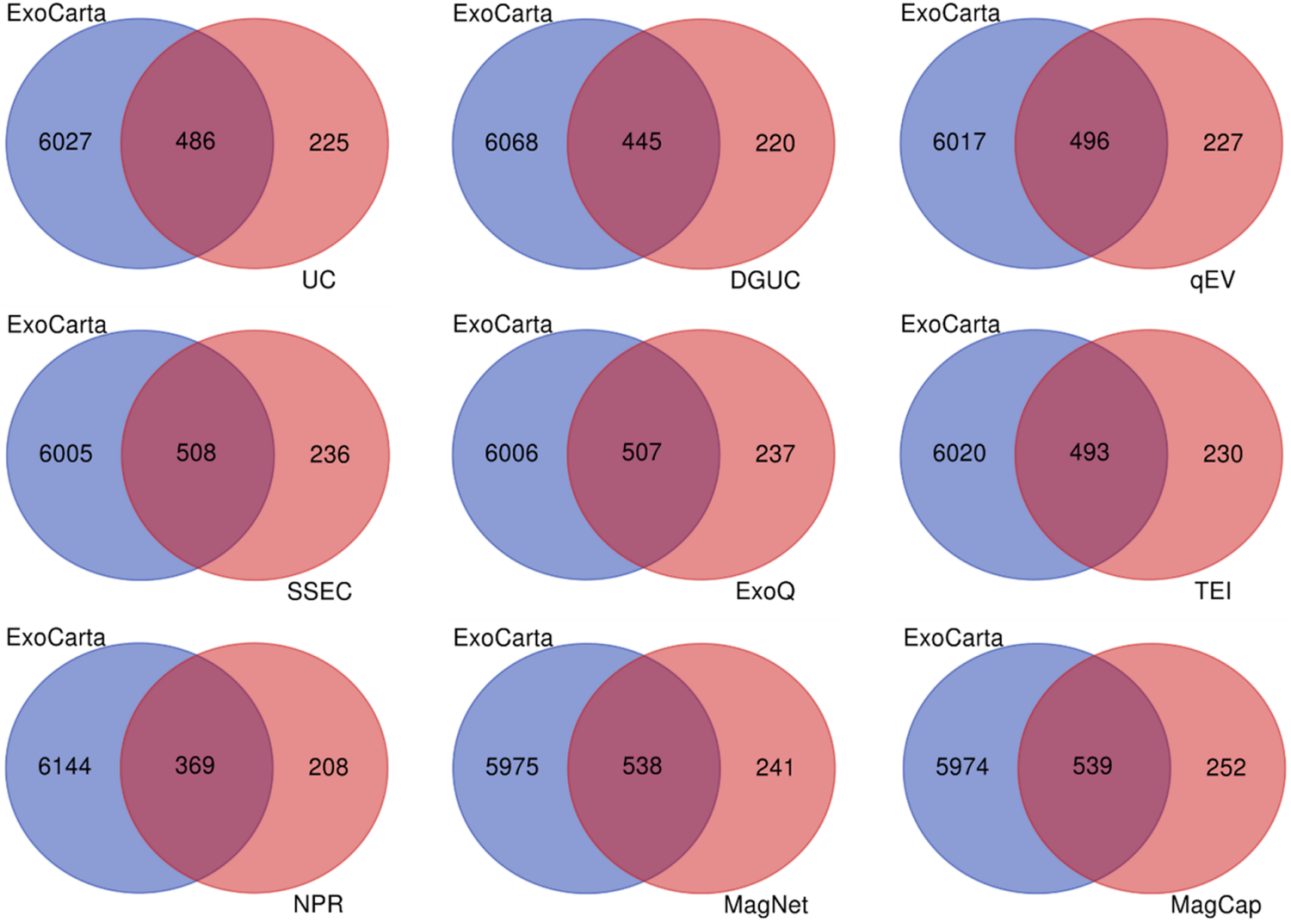
Venn diagram displaying the number of proteins identified in EV preparations based on the EV marker proteins listed in the ExoCarta database.

This comprehensive proteomic analysis also identified proteins unique to the EV-specific isolation methods; e.g., 28 proteins were exclusively identified in MagCap-enriched EVs. Among these, 12 proteins are involved in supramolecular fiber organization, while 8 are linked to the regulation of cell projection organization, suggesting that PS+ EVs play a role in modulating cytoskeleton assembly or cellular movement; in contrast, MagNet-enriched EVs uniquely identified 20 proteins associated with neutrophil degranulation and amino acid biosynthesis.

## 4 Discussion

Since Stahl and Johnstone identified EVs in sheep erythrocyte supernatants in 1983,^41,42^ no universal and standardized techniques have been established for isolating EVs from diverse samples, including cells, tissues, and biofluids. Each method’s efficiency varies depending on the sample type, prompting researchers to seek the most appropriate isolation method for each type of sample. Regardless of the type of biological fluid or the separation method employed, it is critical to understand the characteristics of EV preparations, such as yield, size distribution, purity, and protein composition. Purity is essential when isolating EVs from blood samples due to the high concentration of lipoprotein particles compared to vesicles. Notably, plasma is often preferred over serum for EV isolation, as serum preparation involves clot formation that can release additional EVs from platelets.^43^

Integrating multiple EV isolation methods has achieved plasma EVs with purity and minimal co-isolation of lipoproteins and plasma HAP.^44–46^ In this respect, Karimi et al. demonstrated that sequentially combining DGUC and SEC is pivotal to minimizing contaminants.^13^ While density gradient primarily targets lipoproteins of varying densities, SEC focuses on size differences. By sequentially removing lipoproteins of similar sizes but different densities first, followed by those with similar densities but differing sizes, they achieve markedly pure EVs. However, practical application depends upon sample volume, as smaller volumes can lead to significant EV losses when employing this multimethod strategy. In this study, we focused on evaluating the performance of a single method to enrich EVs from a modest amount of plasma. Our evaluation included the predominantly used commercially available kits for plasma EV isolation, including qEV,^11,35,47^ ExoQ,^35,47,48^ TEI,^35,48,49^ MagNet,^50^ and MagCap.^11,51^ In addition, we included UC and DGUC, two classical EV isolation methods, for comparison. The choice of these methods also considered the sample quantity, cost-effectiveness, time efficiency, specialized equipment requirements, and, most importantly, reproducibility.

All nine methods effectively enrich EVs as evidenced by the enrichment of the canonical EV marker proteins and depletion of albumin and apolipoproteins. UC is a nonspecific and straightforward but time-consuming technique that sediments all plasma vesicles along with protein granules. Based on UC, DGUC further separates particles within a particular density range, primarily enriching small EVs and similar density particles. Therefore, it is expected that DGUC has a lower yield, narrower size distribution, and purer EVs compared to UC, with much reduced albumin and ApoE levels. SEC-based methods nonspecifically separate EV particles based on size. Polymer precipitation works by reducing the solubility of vesicles and similarly sized protein aggregates, which are then separated by low-speed centrifugation.^12^ Additionally, ExoQ coupled with column separation for further purification resulted in a particle yield slightly lower than TEI without much changes in particle size distribution or proteome coverage. The electrostatic interaction and affinity enrichment-based methods, such as NPR, MagNet, and MagCap, are all more selective EV enrichment methods, with MagCap being much more specific because of the affinity of Tim4 protein to PS+ EVs. Because of their specificity, a lower yield is expected, ranging from 1.4 to 4.7 E9 particles. However, the performance of NPR in our hands is not on par with MagNet and MagCap; the latter two are quite comparable in terms of particle purity and proteome coverage. According to the manufacturer, NPR uses a SiC resin, which is typically negatively charged and enriches EVs through electrostatic interactions, opposite the strong anion exchange resin used in MagNet. It is of note that Veerman et al. employed five distinct techniques to isolate EVs from 250 μL of plasma, revealing that the ExoQ method demonstrated superior EV isolation efficiency compared to qEV and DGUC.^51^ Another study corroborated these results, showing that ExoQ yielded a higher particle count than TEI and UC.^52^ These findings diverge from our observations that ExoQ’s yield is higher than UC and DGUC, while slightly lower than TEI and ∼6-fold lower than qEV.

Compared with the universal detection of CD9 in all plasma-derived EV samples, CD81 was only selectively observed in ExoQ, NPR, MagNet, and MagCap methods. The band intensity is much weaker compared to that of CD9, as is the case for the commercial plasma EV standard. A similar trend was observed in some reports.^53,54^ While the isolation method plays a role, we speculate that the diminished detectability of CD81 in general was because of not using a fresh plasma sample, as reported, the CD81 level was decreased in frozen plasma.^55^

This study systematically compared nine EV isolation methods using a streamlined proteomic workflow optimized for throughput, primarily for unbiased assessment of the depletion and enrichment of relatively abundant proteins associated with each EV isolation method beyond the few protein markers measured with Simple Western, rather than for maximal protein coverage. Regarding the number of proteins identified, we used a library-free database search and identified 550–785 proteins from EV samples enriched with different EV isolation methods. At this level, mostly the abundant and medium-abundant proteins were covered, although many low-abundance proteins were also routinely detected. Typically, the protein count was meager without the spectral library, as Veerman et al. reported, who identified 243 proteins by ExoQ, 109 proteins by qEV, and 174 proteins by DGUC in EVs isolated from 250 μL of plasma.^51^ However, Wu et al. identified >4000 plasma proteins, which were simultaneously enriched and digested Plasma EVs using the MagNet bottom-up proteomics protocol with a spectral library and 95 min LC gradient,^50^ while ours was peptide spectral library-free search and a 21 min LC gradient without MagNet on-bead EV digestion. In our hands, doubling the gradient length can result in more protein identifications (from 785 to 1080 for MagNet isolated and digested EV samples; unpublished data). However, it was still less than proteomic analysis from simultaneous EV isolation and on-bead digestion (1219 proteins) using the 21 min gradient, highlighting that proteomic coverage is affected by EV particle isolation and digestion protocols. While it is out of the scope of this work, we found that the freshness of plasma had the most influence on EV plasma proteome coverage. In this respect, using the complete on-bead MagNet proteomics protocol, even with a 21 min gradient and spectra library-free database search, 2492 proteins were identified from freshly collected plasma (unpublished data).

Simple Western spectroscopy confirmed efficient EV recovery across all methods, with CD9, Alix, and TSG101 enrichment and significant depletion of albumin and apolipoproteins. However, proteomics revealed subtle differences beyond these markers, such as method-specific variations in EV-associated proteins; for instance, MagCap and MagNet uniquely enriched cytoskeletal regulators and neutrophil-derived proteins, respectively. Proteomic data aligned with Simple Western but provided broader insights, such as the DGUC preferential clearance of ApoE3-bound lipoproteins.

This study, while comprehensive, has several limitations that must be considered in interpreting its findings. First, using pooled plasma from healthy donors restricts the generalizability of the results, as EV profiles may vary in disease states or across individuals due to factors like age, sex, or metabolic differences. Secondly, the reliance on previously frozen plasma may introduce artifacts, such as EV aggregation or altered surface markers (e.g., reduced CD81 levels), potentially affecting isolation efficiency. Contaminant profiling, though thoroughly investigated for albumin and apolipoproteins, did not fully assess nonvesicular particles, which could confound downstream analyses. Despite these limitations, our findings underscore the utility of proteomics in evaluating EV isolation efficacy beyond canonical markers, highlighting method-specific biases in EV composition and purity.

In conclusion, this study systematically evaluated nine frequently used methods or commercial kits for plasma EV enrichment. All nine methods enriched EVs with a relatively good reproducibility. Although the methods can be categorized into four based on the working principles, methods belonging to the same category do not perform equally. Both UC and DGUC require an ultracentrifugation system, which has low throughput and is time-consuming, particularly for DGUC, which is technically demanding in laying the density gradient. UC produces higher yields but less pure EVs, and DGUC produces purer EVs, albeit with lower yields. The SEC-based qEV and SSEC, as well as polymer precipitation-based ExoQ and TEI, do not require specialized equipment and are easy to perform, resulting in higher yield EVs with relatively less purity, particularly for TEI. The electrostatic interaction-based NPR and MagNet, as well as affinity-based MagCap methods, particularly MagNet and MagCap, are highly effective in enriching EVs with narrow size distribution, high purity, and highest proteome coverage. Although the procedure is long, they are amenable to high-throughput processing of small volume plasma samples, which fit well with biomarker discovery studies.

## Supplementary Material





## Data Availability

The mass spectrometry proteomics data have been deposited to the ProteomeXchange Consortium via the PRIDE^56^ partner repository with the dataset identifier PXD058948. Other data are available upon request from the authors.

## Notes

Full image Simple Western electropherograms of targeted proteins representing canonical EV markers and common contaminants (**Supplementary Figure S1**); hierarchical clustering of identified proteins across different EV isolation methods (**Supplementary Figure S2**) (PDF)
Identified proteins by DIA-NN in each EV isolation method (**Supplementary Table S1**) (XLSX)
