# Supplementary material for: Comprehensive Comparison of Methods for Isolation of Extracellular Vesicles from Human Plasma
Source: J Proteome Res. 2025 May 13;24(6):2956–67. doi: 10.1021/acs.jproteome.5c00149 (PMC12150312; doi:10.1021/acs.jproteome.5c00149)

Comprehensive Comparison of Methods for Isolation of Extracellular Vesicles from Human Plasma

Patil Shivprasad Suresh,[1] Qibin Zhang[1,2]*

[1]Center for Translational Biomedical Research, University of North Carolina at Greensboro, North Carolina Research Campus, Kannapolis, NC 28081, USA.

[2]Department of Chemistry & Biochemistry, University of North Carolina at Greensboro, Greensboro, NC 27402, USA.

Table of Contents

Item	Title	Page Number
**Figure S1**	Full image Simple Western electropherograms of targeted proteins, a) CD9, b) CD81, c) Alix, d) Annexin A2, e) HSPA8, f) TSG101, g) Albumin, h) ApoA1, and i) ApoE3.	**S2-S6**
**Figure S2**	Hierarchical clustering of identified proteins across different EV isolation methods.	**S7**
**Table 1**	Identified proteins by DIA-NN in each EV isolation method.	**XLSX**

Supplementary Figure S1a-i. Simple western full image with electropherogram of targeted proteins, a) CD9, b) CD81, c) Alix, d) Annexin A2, e) HSPA8, f) TSG101, g) Albumin, h) ApoA1, and i) ApoE3.

S1a

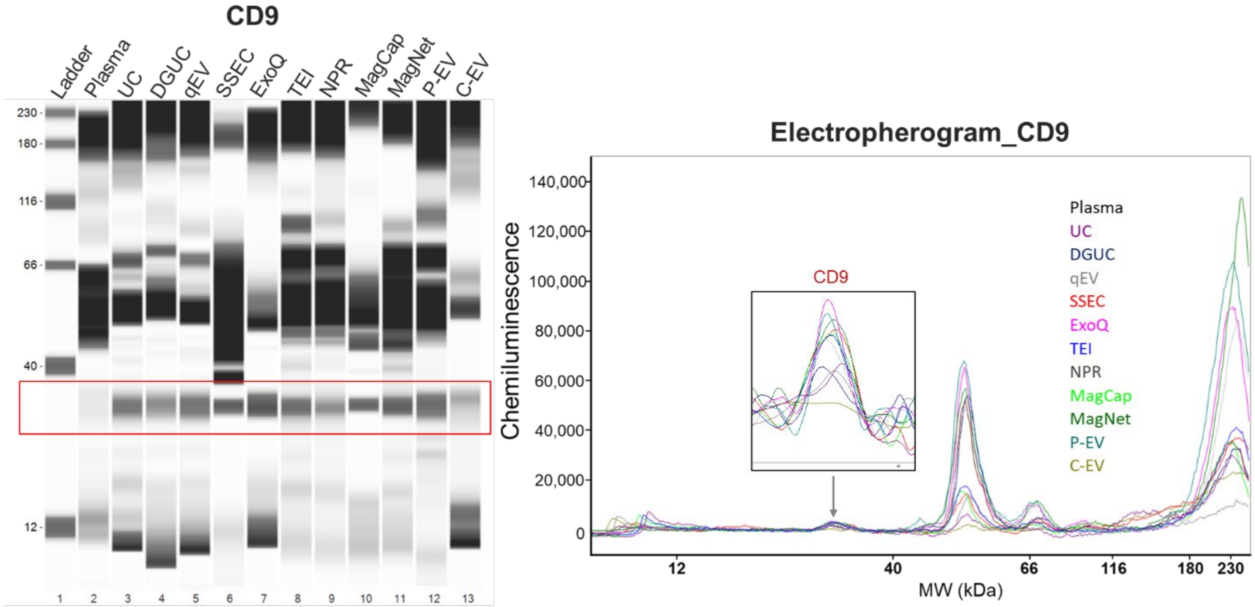

S1b

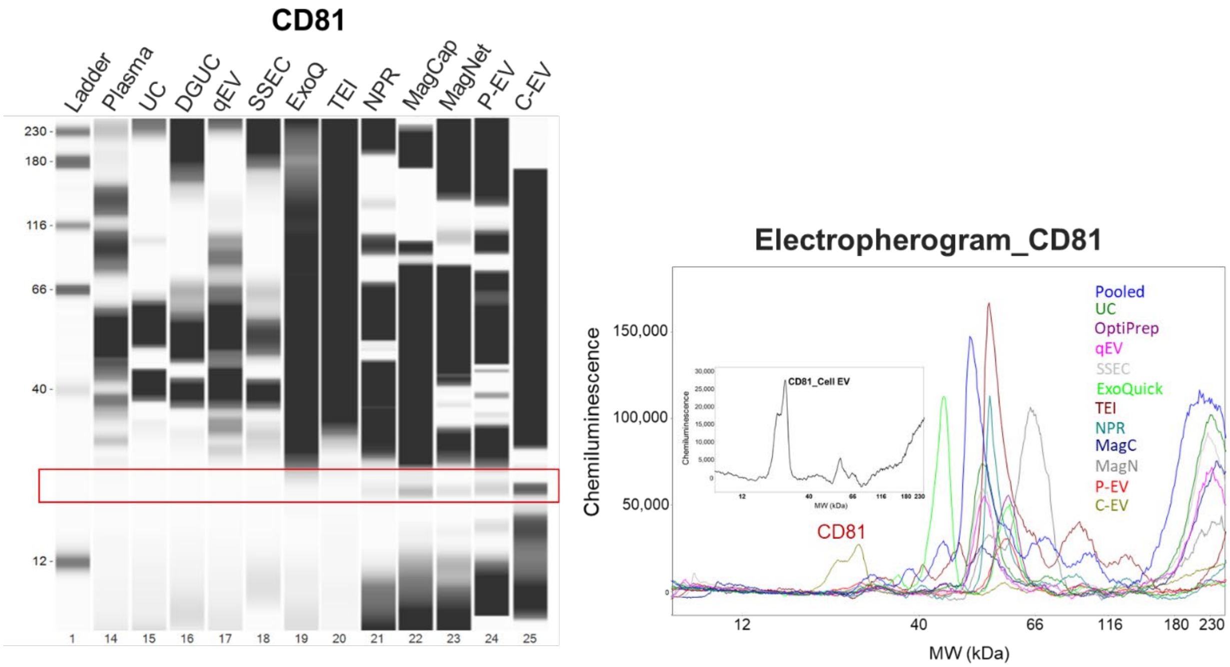

S1c

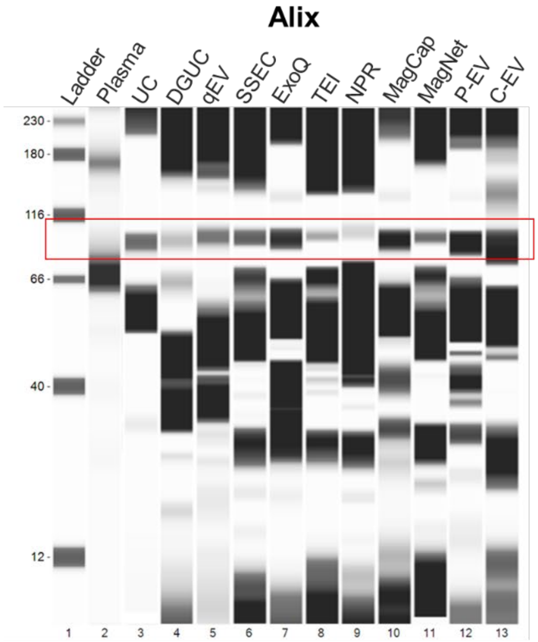

Alix
Ladder
Plasma
UC
DGUC
qEV
SSEC
ExoQ
TEI
NPR
MagCap
MagNet
P-EV
C-EV
230
180
116
66
40
12
1 2 3 4 5 6 7 8 9 10 11 12 13

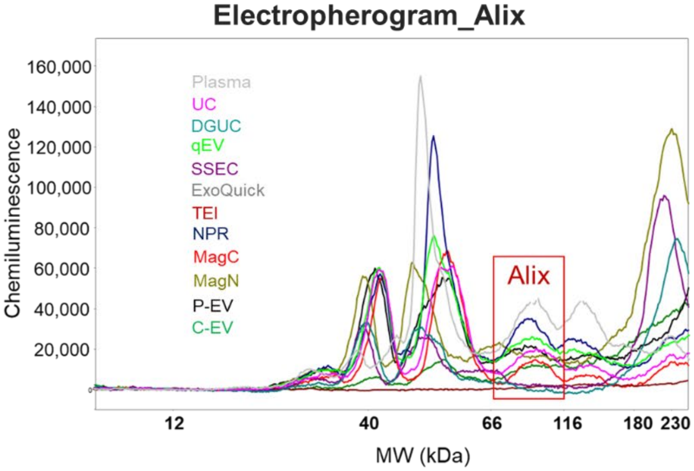

Electropherogram_Alix
Plasma
UC
DGUC
qEV
SSEC
ExoQuick
TEI
NPR
MagC
MagN
P-EV
C-EV
Alix
Chemiluminescence
160,000
140,000
120,000
100,000
80,000
60,000
40,000
20,000
12
40
66
116
180 230
MW (kDa)

S1d

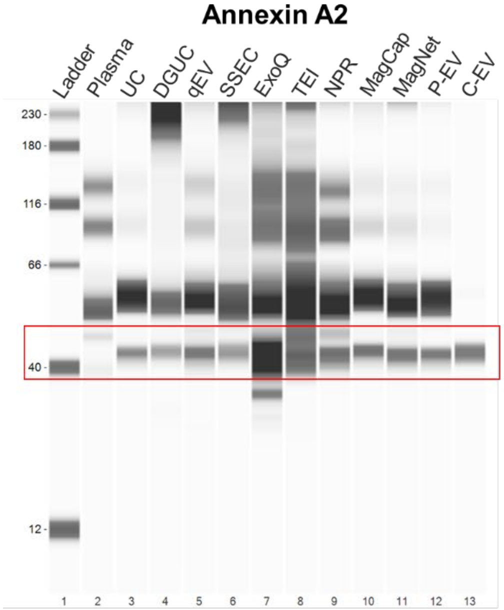

Annexin A2
Ladder
Plasma
UC
DGUC
qEV
SSEC
ExoQ
TEI
NPR
MagCap
MagNet
P-EV
C-EV
230
180
116
66
40
12
1 2 3 4 5 6 7 8 9 10 11 12 13

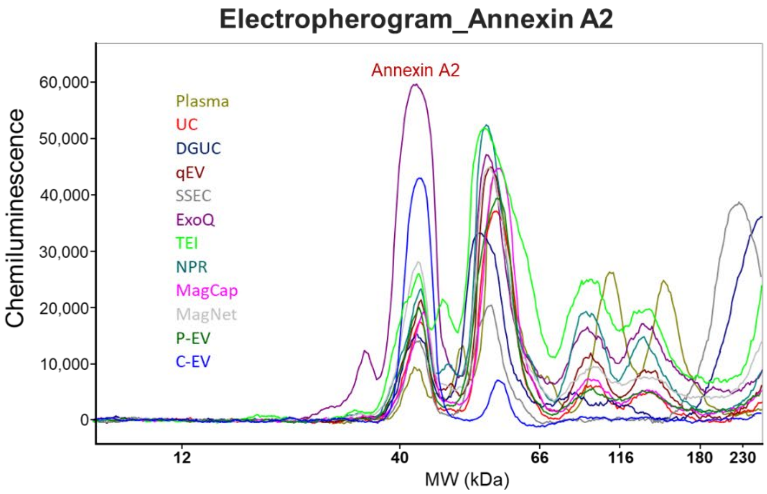

Electropherogram_Annexin A2
Annexin A2
Plasma
UC
DGUC
qEV
SSEC
ExoQ
TEI
NPR
MagCap
MagNet
P-EV
C-EV
Chemiluminescence
60,000
50,000
40,000
30,000
20,000
10,000
0
12
40
66
116
180
230
MW (kDa)

S1e

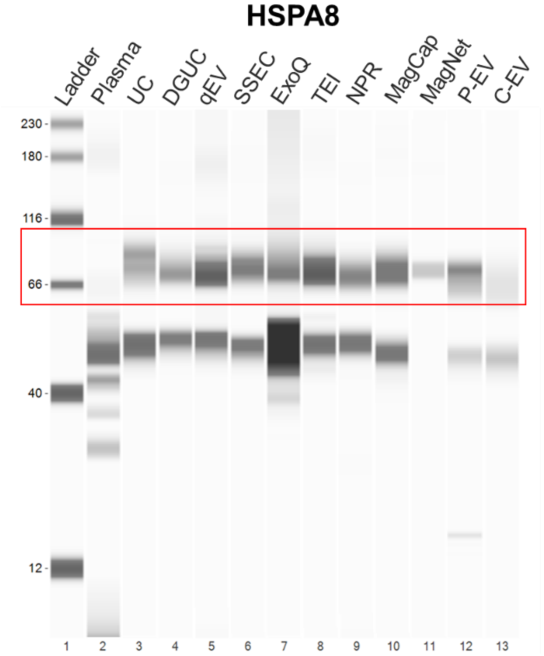

HSPA8
Ladder
Plasma
UC
DGUC
qEV
SSEC
ExoQ
TEI
NPR
MagCap
MagNet
P-EV
C-EV
230
180
116
66
40
12
1 2 3 4 5 6 7 8 9 10 11 12 13

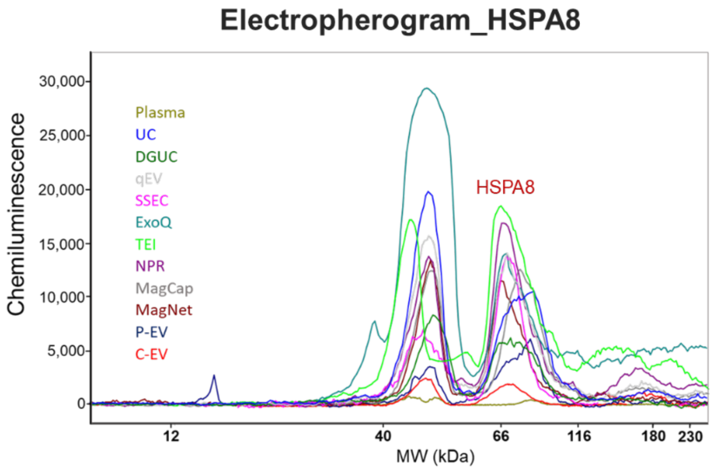

Electropherogram_HSPA8
Chemiluminescence
30,000
25,000
20,000
15,000
10,000
5,000
0
Plasma
UC
DGUC
qEV
SSEC
ExoQ
TEI
NPR
MagCap
MagNet
P-EV
C-EV
HSPA8
12
40
66
116
180
230
MW (kDa)

S1f

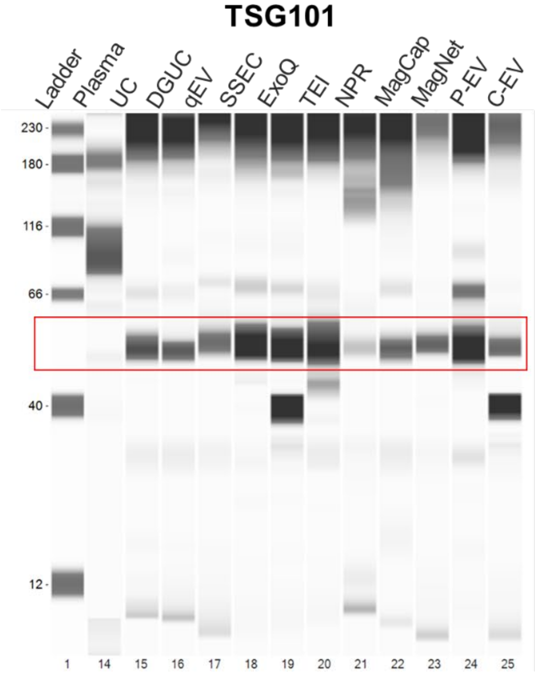

TSG101
Ladder
Plasma
UC
DGUC
qEV
SSEC
ExoQ
TEI
NPR
MagCap
MagNet
P-EV
C-EV
230
180
116
66
40
12
1 14 15 16 17 18 19 20 21 22 23 24 25

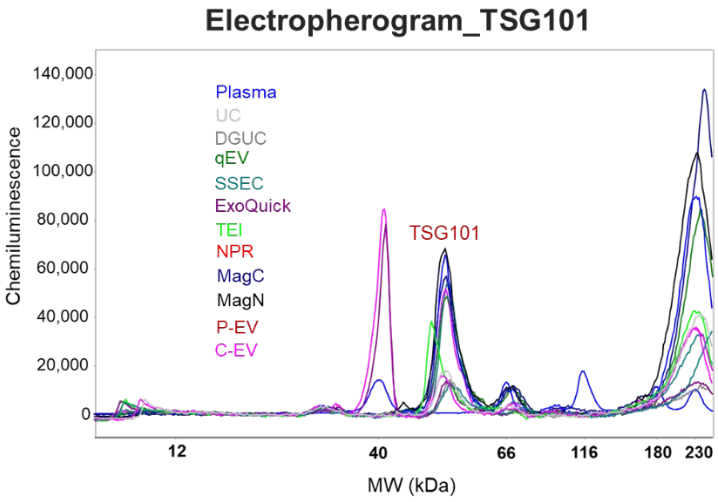

Electropherogram_TSG101
Chemiluminescence
140,000
120,000
100,000
80,000
60,000
40,000
20,000
0
Plasma
UC
DGUC
qEV
SSEC
ExoQuick
TEI
NPR
MagC
MagN
P-EV
C-EV
TSG101
12
40
66
116
180
230
MW (kDa)

S1g

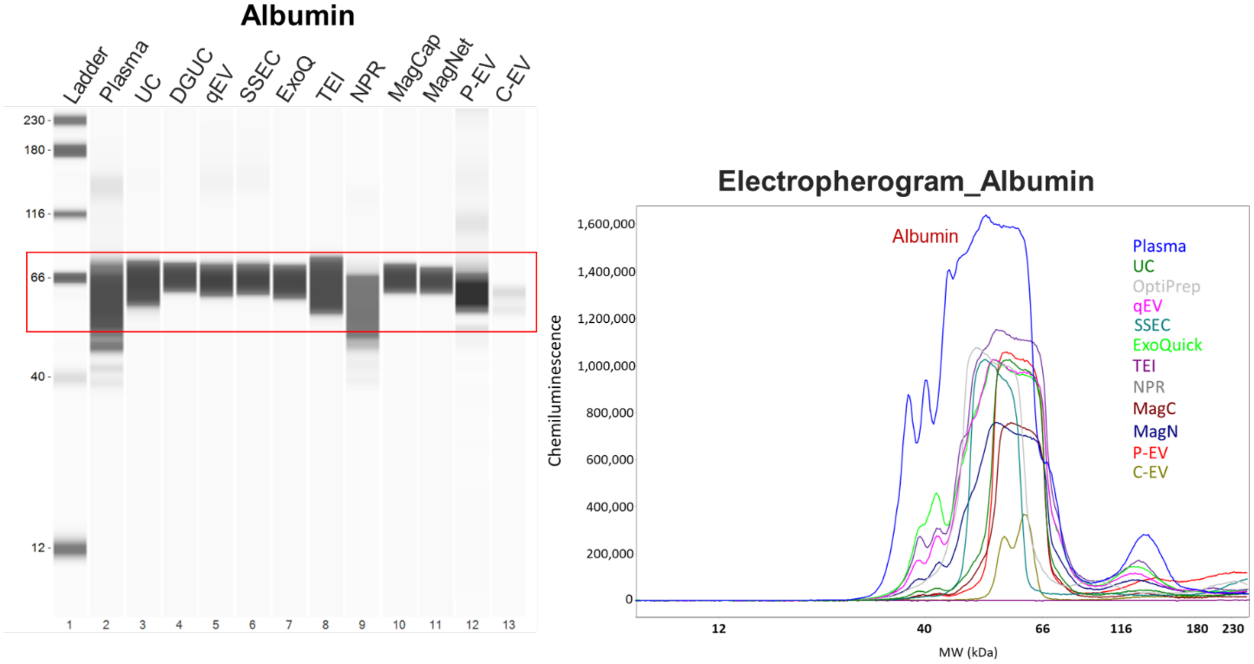

Albumin
Ladder
Plasma
UC
DGUC
qEV
SSEC
ExoQ
TEI
NPR
MagCap
MagNet
P-EV
C-EV
230
180
116
66
40
12
1 2 3 4 5 6 7 8 9 10 11 12 13
Electropherogram_Albumin
Albumin
Plasma
UC
OptiPrep
qEV
SSEC
ExoQuick
TEI
NPR
MagC
MagN
P-EV
C-EV
Chemiluminescence
1,600,000
1,400,000
1,200,000
1,000,000
800,000
600,000
400,000
200,000
0
12
40
66
116
180
230
MW (kDa)

S1h

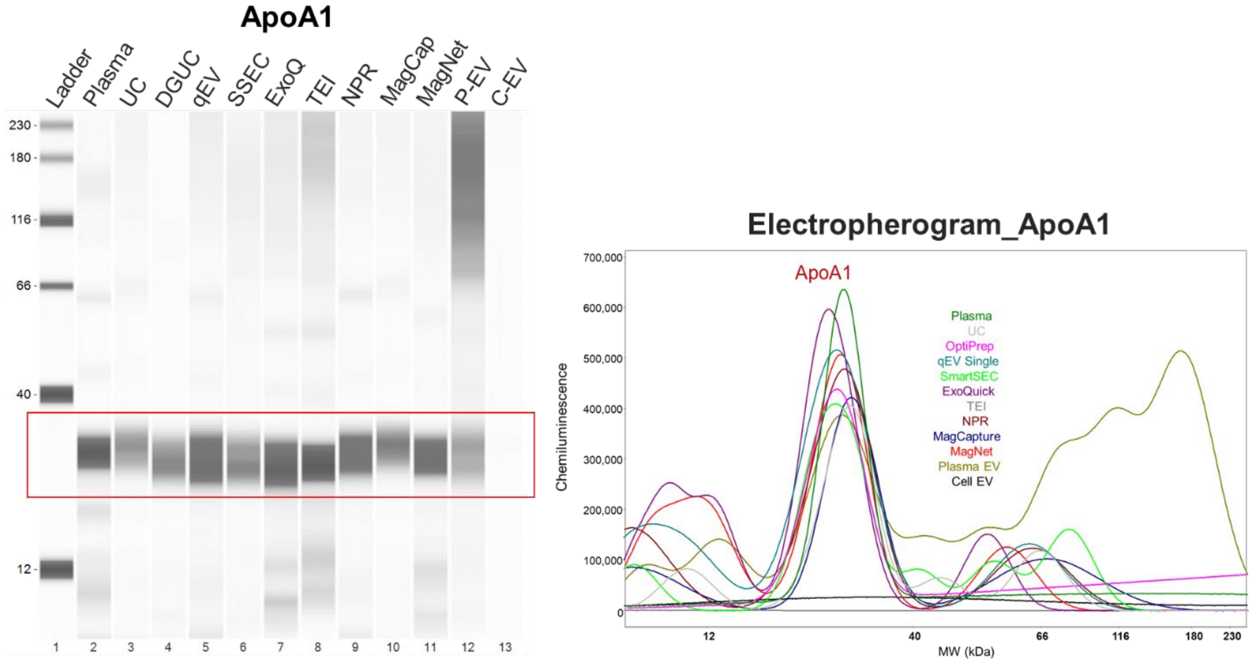

ApoA1
Ladder
Plasma
UC
DGUC
qEV
SSEC
ExoQ
TEI
NPR
MagCap
MagNet
P-EV
C-EV
230
180
116
66
40
12
1 2 3 4 5 6 7 8 9 10 11 12 13
Electropherogram_ApoA1
ApoA1
Plasma
UC
OptiPrep
qEV Single
SmartSEC
ExoQuick
TEI
NPR
MagCapture
MagNet
Plasma EV
Cell EV
Chemiluminescence
700,000
600,000
500,000
400,000
300,000
200,000
100,000
0
12
40
66
116
180
230
MW (kDa)

S1i

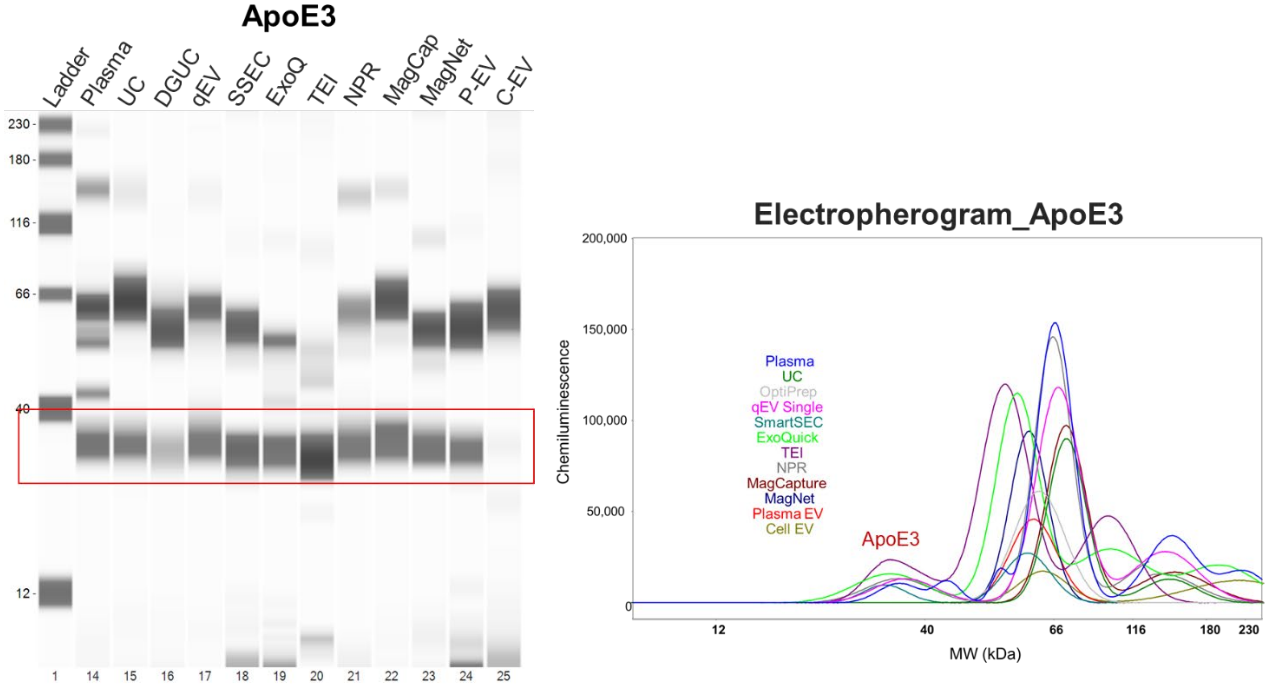
ApoE3
Ladder
Plasma
UC
DGUC
qEV
SSEC
ExoQ
TEI
NPR
MagCap
MagNet
P-EV
C-EV
230
180
116
66
40
12
1
14
15
16
17
18
19
20
21
22
23
24
25
Electropherogram_ApoE3
200,000
150,000
100,000
50,000
0
Chemiluminescence
Plasma
UC
OptiPrep
qEV Single
SmartSEC
ExoQuick
TEI
NPR
MagCapture
MagNet
Plasma EV
Cell EV
ApoE3
12
40
66
116
180
230
MW (kDa)

Supplementary Figure S2. Hierarchical clustering of identified proteins across different EV isolation methods.

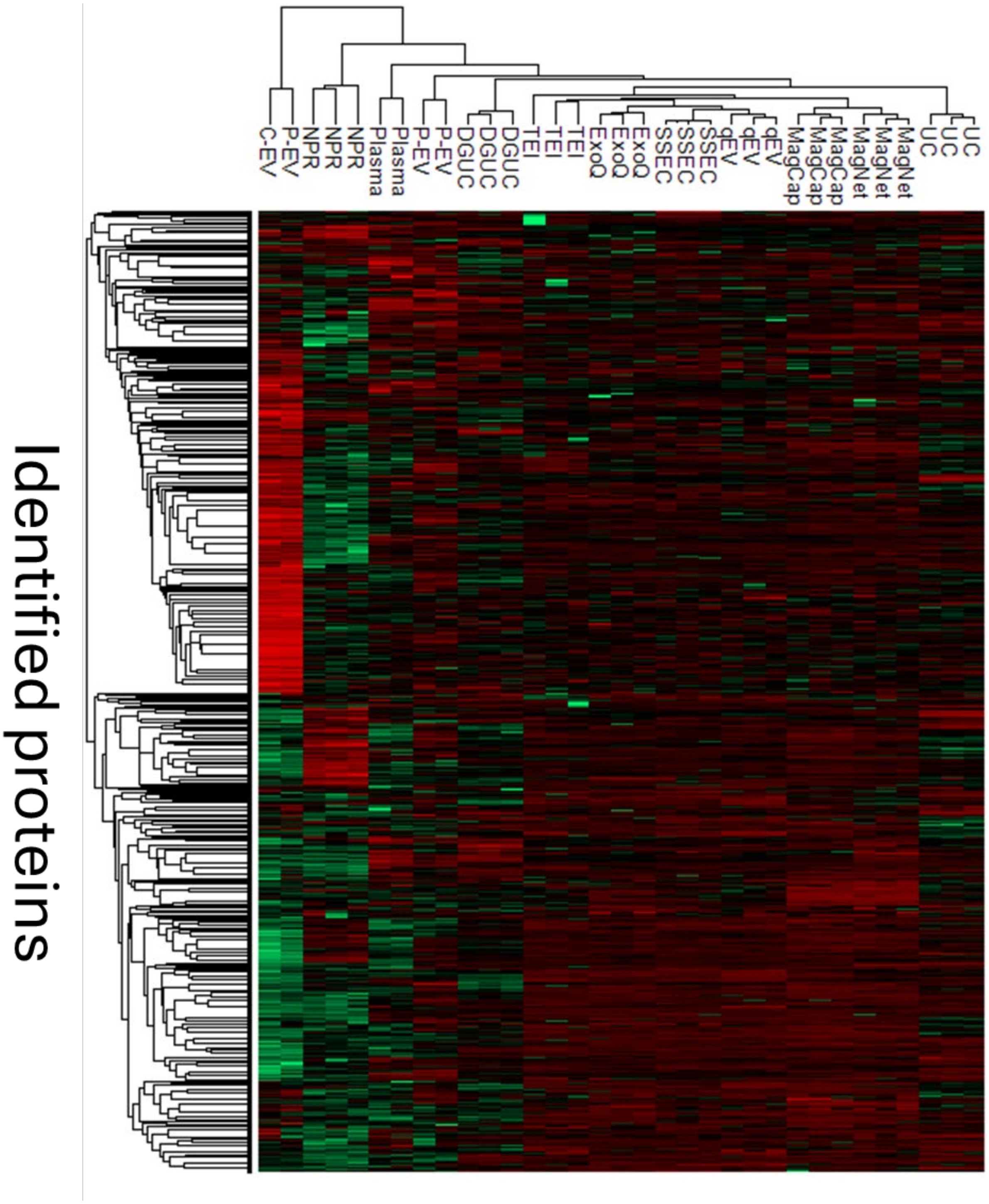

Supplement: Supplementary file 1 [file pr5c00149_si_001.pdf]
